# The effects of vocal emotions and emotional context on the neural tracking of speech envelopes and listeners’ vigilance states

**DOI:** 10.3389/fnhum.2026.1692628

**Published:** 2026-05-08

**Authors:** Teagan Esme Esther, Liron Shlesinger, Fernando Llanos

**Affiliations:** 1 Department of Linguistics, The University of Texas at Austin, Austin, TX, United States; 2 Department of French and Italian, The University of Texas at Austin, Austin, TX, United States

**Keywords:** alpha desynchronization, alpha suppression, amplitude modulation, arousal, EEG, emotional speech, speech envelope, speech processing

## Abstract

**Introduction:**

High-arousal emotional speech, such as angry and happy speech, is characterized by changes in signal amplitude that can substantially alter the temporal structure of the speech signal. In this EEG study, we investigated how these acoustic changes, and the structure of the preceding emotional context, influence neural tracking of temporal speech patterns, as well as alpha-band desynchronization associated with vigilance states in listeners.

**Methods:**

EEGs were recorded from 30 adult native speakers of American English while they listened to angry, happy, or neutral spoken sentences presented either in a randomized order or blocked by emotion. To ensure sustained attention, participants answered occasional questions about sentence content.

**Results:**

Angry speech elicited stronger alpha desynchronization than neutral and happy speech when stimuli were blocked by emotion but not when stimuli were fully randomized. In contrast, neural tracking of amplitude modulation patterns was more robust for neutral speech compared to happy or angry speech across both stimulus presentation contexts. When neural tracking was controlled for stimulus differences in amplitude variability, angry speech, which conveyed greater amplitude variability, was more faithfully tracked than both happy and neutral speech across stimulus presentation contexts.

**Discussion:**

Our findings suggest that tonic modulations of alpha power are more sensitive to prolonged emotional context than to transient changes in speaker emotion. Furthermore, we found that emotional speech robustly modulates listeners’ vigilance, particularly following prolonged exposure to a single emotion, while exerting a limited influence on the neural encoding of amplitude modulation, which is primarily dominated by bottom-up amplitude variability in the acoustic signal.

## Introduction

Vocal emotions play an important role in human communication because they allow us to express and perceive mental states. Emotional speech is characterized by vocalizations (Coutinho and Dibben, 2013; Frick, 1985; Laukka et al., 2005; Lieberman and Michaels, 1962) that can alter the spectro-temporal structure of speech signals (see the subsections below). Understanding how these vocalizations influence both speech processing and listeners’ mental states is critical for the development of naturalistic models of spoken communication. Crucially, these influences may depend on the structure of the preceding emotional context, namely whether emotional expressions are sustained over time or rapidly intermixed. In the present study, we investigated how vocal emotions and the emotional context in which they occur influence (1) the neural tracking of amplitude modulation, which provides an index of continuous speech processing, and (2) alpha desynchronization, which is associated with higher vigilance in listeners. Together, these two neural markers provide a comprehensive account of how speakers’ emotions and their effects on listeners’ internal processing states influence the encoding of temporal speech patterns that are relevant for speech intelligibility.

### Perception of emotional speech

Emotional speech involves systematic modifications of the voice that can significantly alter the temporal structure of speech signals. Anger is typically expressed with increased amplitude, higher fundamental frequency (F0), and faster articulation rates, although under some conditions it can also be realized with slower tempo and longer utterance duration (Johnstone and Scherer, 2000; Scherer, 1986; Scherer and Banse, 1996). Sadness is often associated with reduced intensity, lower pitch, and slower tempo, while happiness and fear have been linked to greater pitch variability and changes in spectral tilt (Juslin and Laukka, 2003; Laukka et al., 2005). Notably, these patterns reflect both physiological constraints on vocal articulation under emotional arousal and communicative conventions that enable speakers to convey affective intent.

Listeners are highly sensitive to emotional speech cues and basic vocal emotions can be identified across languages and cultures with above-chance accuracy, although recognition rates vary across categories, with anger and happiness typically identified more reliably than fear or disgust (Pell et al., 2009; Scherer et al., 2001). Beyond emotion recognition, emotional prosody has also been shown to influence the perception of speech patterns, improving or hindering word recognition under adverse listening conditions (Alexander and Llanos, 2025; Cohn et al., 2021; Dupuis and Pichora-Fuller, 2014; Gordon and Ancheta, 2017). These effects, whether facilitatory or disruptive, depend on both the emotional content and task demands. In auditory lexical decision tasks, for instance, emotionally congruent vocal and lexico-semantic cues can facilitate lexical and emotional processing (Feeny et al., 2019; Gao et al., 2022; Nygaard and Lunders, 2002; Pell et al., 2011; Wurm et al., 2001). Conversely, when the task requires recognizing the vocal identity of a given speaker, the presence of task-irrelevant emotional cues can negatively interfere with the processing of speaker-specific information (Aue et al., 2011).

### Neural processing of emotional speech

Prosodic cues associated with emotion have been shown to recruit auditory cortices as well as brain regions implicated in affective evaluation, such as the amygdala and orbitofrontal cortex (Ethofer et al., 2006; Grandjean et al., 2005; Schirmer and Kotz, 2006). Mechanistically, ERP studies have demonstrated an early temporal dissociation in the detection of emotional versus neutral speech, with differences in the time course of ERPs emerging within the first 200 ms after stimulus onset (Jessen and Kotz, 2011; Paulmann and Kotz, 2008a). The detection of emotional speech features is typically followed by a later integration window, after 300 ms post-stimulus onset, during which expected and perceived emotions are integrated (Kanske et al., 2011; Paulmann and Kotz, 2008b; Zora et al., 2020).

In addition to the effects of emotional speech on ERPs, emotional prosody can also modulate neural oscillations underlying listeners’ mental states. In particular, alpha-band activity (8–12 Hz) has been linked to attentional control and emotional states. Here, alpha desynchronization is generally interpreted as reflecting increased vigilance and the reallocation of attentional resources to salient or motivationally relevant stimuli. Consistent with this view, emotionally arousing inputs, including fearful or angry faces, affective sounds, and emotionally charged words, are often associated with reduced alpha power relative to neutral stimuli (Aftanas et al., 2002; Güntekin and Basar, 2007; Knyazev, 2007). Within the auditory domain, emotional prosody modulates alpha power, with high-arousal vocal expressions eliciting greater desynchronization than neutral speech (del Giudice et al., 2016). Alpha modulation has further been interpreted as a gating mechanism, whereby oscillatory activity implements functional inhibition of task-irrelevant systems (Foxe and Snyder, 2011; Klimesch, 2012), thereby facilitating the allocation of resources toward task-relevant processing.

### Neural tracking of amplitude modulation

Previous neuroscientific research on emotional speech has primarily investigated the identification of speakers’ emotions and their effects on listeners’ affective states. Consequently, our current understanding of how speakers’ emotions influence the neural coding of specific speech features is more limited. To address this research gap, we investigated the effects of emotional speech on the neural tracking of amplitude modulation. The recognition of speech utterances relies on both amplitude modulation patterns (such as the amplitude or speech envelope) and frequency modulation patterns (including F0, harmonics, and formants), which are differentially encoded within the auditory system. However, there is evidence that slow amplitude modulation (especially modulation frequencies between 1 and 8 Hz) is more critical for speech intelligibility than frequency-specific modulation patterns (Elliott and Theunissen, 2009; Houtgast and Steeneken, 1985). While listeners can still recognize speech units (syllables, words, and phrases) with minimal temporal fine structure (Rosen, 1992; Shannon et al., 1995; Stilp et al., 2010), temporal distortions of syllables and words can drastically impair speech intelligibility and slow down processing (Drullman, 1995; Healy et al., 2005).

The processing of amplitude modulation patterns in the temporal cortex is achieved through a temporal code (Alexandrou et al., 2020; Ding and Simon, 2014; Peelle and Davis, 2012). Single unit recording studies in non-human models (Wang, 2007; Wang et al., 2003) have shown that neurons in the primary auditory cortex phase-lock to changes in stimulus amplitude to encode an analogous version of the amplitude envelope. In humans, the neural tracking of amplitude modulation has been examined using multiple technologies, such as electrocorticography (ECoG) (e.g., Nourski and Brugge, 2011), magnetoencephalography (MEG) (e.g., Ding et al., 2016), and EEG (e.g., Di Liberto et al., 2015). This research demonstrates that the neural tracking of amplitude modulation in connected speech can be influenced by a diversity of factors, such as attention (Lakatos et al., 2008; Obleser and Kayser, 2019), language experience (Reetzke et al., 2020; Song and Iverson, 2018), or speech intelligibility (Peelle et al., 2013; Peelle and Davis, 2012; Vanthornhout et al., 2018). These findings indicate that the neural tracking of amplitude modulation reflects more than just the sensory transduction of temporal attributes. Indeed, computational neuroscience work (Di Liberto et al., 2015; Huth et al., 2016) has shown that the prediction of brain oscillations and hemodynamic responses from amplitude envelope oscillations improves when abstract linguistic features are incorporated as regressors in the model. This finding indicates that the neural tracking of amplitude modulation in speech signals can potentially provide insight into the extraction of discrete primitives that are linguistically relevant.

### The present study

Given that amplitude modulation is critical for intelligibility and that it can be substantially altered by speakers’ emotions, we aimed to examine the effects of emotional speech on the neural tracking of amplitude modulation patterns in continuous speech. High-arousal vocal emotions such as angry and happy speech exhibit greater envelope variability that could potentially reduce tracking fidelity relative to neutral speech. Consistent with this hypothesis, previous research has reported a less faithful neural representation of speech signals when their temporal structure is more irregular or less predictable (Falk et al., 2017; Kayser et al., 2015). At the same time, high-arousal emotional stimuli are expected to elicit stronger emotional responses (del Giudice et al., 2016) that could potentially enhance attention and sensory gain, facilitating the gating of sensory inputs (Aston-Jones and Cohen, 2005; Ghacibeh et al., 2006; Mather and Sutherland, 2011; Sara, 2009). Thus, whether the amplitude envelope of high-arousal emotional speech is tracked more or less faithfully than that of neutral speech likely depends on the balance between the effects of input processing demands due to bottom-up acoustics and the effects of speakers’ emotions on listeners’ processing states.

Additionally, we investigated the effects of emotional context by comparing the effects of prolonged exposure to a single emotional tone with mixed exposure to multiple emotions. Previous neuroscientific studies have typically relied on isolated emotional utterances presented in random order. As a result, little is known about how sustained exposure to a single emotional tone (e.g., several minutes of angry, happy, or neutral speech) influences alpha-band power and neural tracking relative to intermixed emotional exposure (e.g., several minutes of speech varying at random between angry, happy, and neutral speech). Continuous exposure to stimuli characterized by a single emotion may induce mood-like states, leading to emotion-level differences in alpha desynchronization or neural tracking that are absent when emotions are presented in unpredictable ways. This hypothesis is supported by evidence showing that sustained exposure to positive or negative emotional stimuli can elicit prolonged changes in neural and behavioral responses, persisting after the exposure period (Barazzone and Davey, 2009; Halberstadt et al., 1995). Such mood-related changes appear to be independent of specific stimulus features, as mood does not shift spontaneously or in response to a single, brief stimulus presentation (Beedie et al., 2005; Brewer et al., 1980; Egidi and Nusbaum, 2012; Kučera and Haviger, 2012).

To address these hypotheses, we recorded continuous EEG while 30 native English speakers listened to multiple repetitions of 10 semantically neutral sentences produced with angry, happy, or neutral prosody. Participants performed a word recall task to ensure consistent levels of engagement throughout the entire session. To keep the duration of the whole experiment under 2 h, half of the participants (*N* = 15) were assigned to a blocked condition, in which stimulus presentation was blocked by emotion, and the other half was assigned to a randomized condition, in which stimulus presentation was randomized across emotions. Each spoken sentence was presented 36 times to increase the signal-to-noise ratio of the corresponding average response. This design allowed us to examine how emotional context (blocked vs. randomized exposure) influenced both alpha desynchronization and neural tracking of amplitude modulation.

To characterize the perceptual properties of the stimulus set, participants completed a behavioral task prior to EEG recording in which they rated each audio for valence (pleasantness) and arousal (intensity) and categorized the intended emotion using a seven-label basic emotion framework (anger, disgust, fear, happiness, sadness, surprise, neutral), with presentation order varying by EEG condition.

## Methods

The study was approved by the Institutional Review Board (IRB) of the University of Texas at Austin. Anonymized research data and code are available for public consultation on the following online repository: https://osf.io/j89bw/.

### Participants

Research data were collected in the University of Texas at Austin area from 30 young adults who were native speakers of American English (6 men, 23 women, 1 unspecified; age: *M* = 21.30, SD = 3.45, range = 18–33). Participants provided written consent prior to their participation in the experiment and reported no history of hearing impairment, neurodevelopmental disorders, nor psychiatric disorders. Participants were compensated at a rate of $15 per hour, for approximately 1.5 h.

### Stimuli

**Figure 1:**
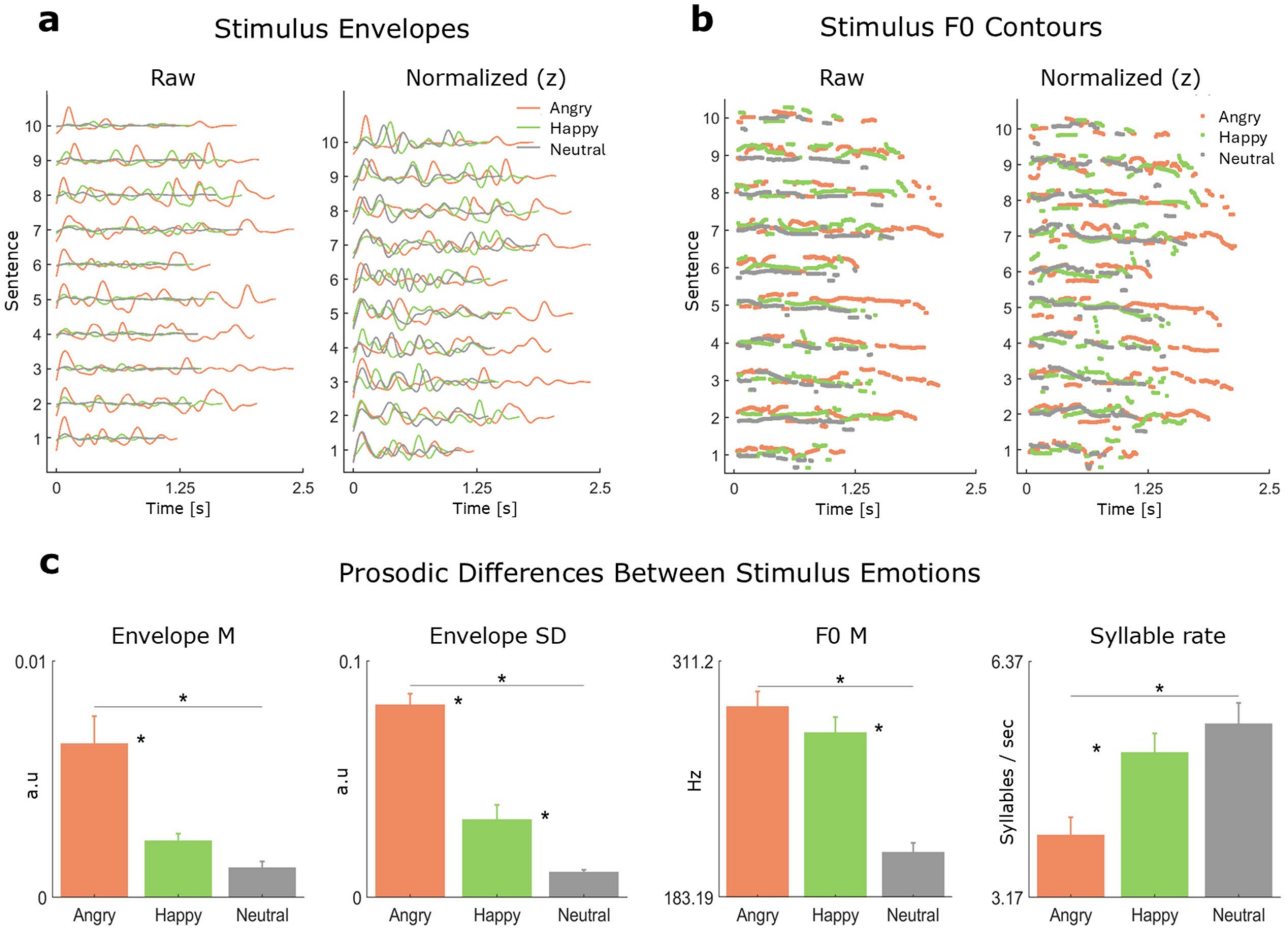
Acoustics. **(a)** Speech amplitude envelopes in raw (left) and *z*-scored (right) units. **(b)** F0 contours in raw (left) and *z*-scored (right) units. **(c)** Mean and standard error mean (SEM) of the four prosodic markers influenced by emotional speech context (M = mean, SD = standard deviation). F0 SD did not significantly vary across emotions (see Table 1 in the *Results* section below) and is thus excluded from this figure. Significant differences (*p* < 0.05) are indicated with a single asterisk (*).

Speech stimuli consisted of 30 emotional speech audios downloaded from the CREMA-D dataset (Cao et al., 2014). This dataset includes speech utterances produced across six basic emotions (happy, sad, angry, fearful, disgusted, and neutral) by 91 actors in the context of 12 semantically neutral sentences (Russ et al., 2008). The CREMA-D dataset also includes a subset of perceptual categorization responses obtained via crowdsourcing from 2,443 listeners instructed to recognize the intended emotions of the audios. Our stimulus set consisted of 10 semantically-neutral spoken sentences, each produced by the same female speaker (speaker code: 1089) across three emotions (angry, happy, neutral), yielding a total of 30 unique audios. Using a single speaker allowed us to isolate prosodic variation while minimizing variability due to talker-specific spectral characteristics. The selected speaker was associated with higher-than-average emotion recognition scores. The amplitude envelopes and F0 contours of the speech stimuli are shown in Figures 1a,b.

### Acoustic analyses

We conducted acoustic analyses to identify major prosodic differences between emotional speech categories. For each audio, we measured: (1) the mean and (2) standard deviation of the speech envelope; (3) the mean and (4) standard deviation of the F0 contour; and (5) the syllable rate. Amplitude envelopes were computed as the absolute value of the Hilbert transform of the speech waveform. The resulting envelope signal was resampled to the sampling rate of the EEG (500 Hz) and band-pass filtered (zero-phase, 4th-order Butterworth) between 1 and 8 Hz to isolate amplitude modulation frequencies most relevant for speech intelligibility (Houtgast and Steeneken, 1985; Stilp et al., 2010). Because this frequency range lies well below the EEG sampling rate, the resampling procedure minimizes the risk of temporal distortions.

F0 contours were extracted in Praat V 6.1.38 (Boersma and Weenink, 2018) using the autocorrelation method (pitch floor: 75 Hz; pitch ceiling: 400 Hz) in keeping with previous studies of emotional speech prosody (Hammerschmidt and Jürgens, 2007; Juslin and Laukka, 2001; Sauter et al., 2010). Syllable rate was calculated manually for each sentence as the inverse of the mean syllable duration (Scherer, 2013; Scherer and Banse, 1996), yielding a clear and interpretable measure of speech rate (Buller, 2005).

To assess whether emotional speech context influenced prosodic markers, we conducted parametric (ANOVA) or non-parametric (Kruskal–Wallis) hypothesis testing, as appropriate. When data met parametric assumptions, analyses were conducted using ANOVA followed by *post hoc* comparisons. When assumptions were violated, Kruskal–Wallis tests were used, followed by rank-based multiple comparisons implemented via MATLAB’s *multcompare* function. For non-parametric analyses, post hoc comparisons operated on mean ranks and did not assume normality or homoscedasticity. In all cases, post hoc pairwise comparisons were corrected for multiple testing using the Tukey–Kramer procedure, as implemented in MATLAB’s *multcompare* function.

### Behavioral procedures

Prior to the EEG session, each participant completed a Qualtrics survey to rate each of the speech stimuli for emotional valence and arousal using the Self-Assessment Manikin (SAM) rubric (Bradley and Lang, 1994; Verma and Tiwary, 2017). This rubric includes nine graphically illustrated levels of valence and arousal ranging from lower arousal/valence (level 1) to higher arousal/valence (level 9). The SAM rubric has been used to rate levels of emotional speech in previous related work (Chen et al., 2019; Grimm et al., 2007; Guntupalli et al., 2012; Siegert et al., 2011). Before moving to the next audio, participants were also asked to label the emotion intended by the speaker as one of the basic six emotion labels included in the CREMA-D dataset plus one additional label, “surprise,” which is common in the literature (Hammerschmidt and Jürgens, 2007; Lausen and Hammerschmidt, 2020; Monnot et al., 2003; Paulmann and Kotz, 2008a; Sauter et al., 2010). For participants assigned to the randomized context, sentence and emotion were fully randomized across trials. For participants assigned to the blocked context, emotional content was presented in a more predictable manner, such that each sentence was heard sequentially in all three emotional states before proceeding to the next sentence (fixed order). This design avoided fully blocking by emotion, which could have reduced task engagement due to repeated exposure to the same emotion across extended periods.

To assess the correspondence between intended and perceived emotional features, individual ratings of arousal and valence were independently modeled with two linear mixed-effects equations. Each model equation was coded in R as follows: rating ~ emotion * stimulus presentation + (emotion | participant) + (1 | sentence), where *emotion* denotes the intended vocal emotion. The equation included: two fixed effects by emotion (factor levels: angry, happy, neutral) and stimulus presentation order (factor levels: fixed, randomized); by-participant random intercepts and random slopes for emotion; and by-sentence random intercepts. Models were fitted using the *lme4* package (Bates et al., 2015), and post-hoc pairwise comparisons were conducted using the *emmeans* (Lenth et al., 2019) package with Tukey adjustments for multiple comparisons.

To ensure the three intended emotions were properly perceived, emotion categorization responses were coded as ‘1’ (correct) and ‘0’ (incorrect), respectively, and modeled with the following logistic mixed-effects equation in R: accuracy ~ emotion * stimulus presentation + (1 | participant) + (1 | sentence), where *accuracy* denotes the dependent variable (correct, incorrect) and *emotion* denotes the intended vocal emotion. The equation included: two fixed effects by emotion (factor levels: angry, happy, neutral) and stimulus presentation order (factor levels: fixed, randomized); by-participant random intercepts; and by-sentence random intercepts. By-participant random slopes were excluded because the logistic equation failed to converge when these terms were included. Post-hoc pairwise comparisons were conducted using the *emmeans* package with Tukey adjustments for multiple comparisons.

Additionally, we conducted a chi-square test of independence to determine whether the distribution of participants’ responses across the seven basic categories varied across stimulus presentation orders (fixed vs. randomized).

### EEG session

During the EEG session, participants listened to 36 repetitions of each audio (*N* = 30), yielding a total of 1,080 trials, with an inter-stimulus interval of 500 ms. The number of repetitions was determined in a pilot session to ensure a robust signal-to-noise ratio in the averaged neural response. To keep the duration of the EEG session manageable, the stimulus presentation context (blocked vs. randomized) was implemented as a between-subjects manipulation, such that each participant completed only one presentation context. In the blocked context, stimulus presentation was blocked by emotion and audios were presented in pseudo-random order within each block, avoiding consecutive repetitions of the same sentence. To prevent any emotional carryover effects due to mood-level changes induced by prolonged exposure to angry or happy speech (Barazzone and Davey, 2009; Halberstadt et al., 1995), the neutral block was presented first, followed by the angry and happy blocks, which were counterbalanced across participants.

While there is evidence that the neural tracking of amplitude modulation does not necessarily deteriorate as the duration of the EEG session increases (Cabral-Calderin and Henry, 2022), factors such as listening effort, attention, and fatigue can still play a role (Hjortkjær et al., 2020; Obleser and Kayser, 2019; Vanthornhout et al., 2019). To minimize potential effects of attention and fatigue on neural tracking, we included short rest periods between each pair of blocks. Additionally, participants were occasionally presented with random yes/no questions about the presence of a specific word in the immediately preceding sentence (e.g., “Did the last sentence you heard include the word ‘jacket’?”). In both stimulus presentation contexts, word detection questions were interpolated randomly within segments of 15 trials, resulting in 24 questions per block. Participants performed at ceiling, achieving 99.10% accuracy in the blocked context and 98.88% accuracy in the randomized context.

Speech stimuli were binaurally delivered using a pair of electrically shielded Etymotic ER3C insert earphones. Word detection questions were displayed using an electrically shielded VIEWPixx monitor (VPixx Technologies). Participants were instructed to answer as soon as possible by pressing the corresponding button (9 “yes”/0 “no”) on a keyboard. EEGs were acquired using 32 actiCAP snap electrodes (Brain Products GmbH) placed on one elastic cap (EasyCap GmbH) with a common ground at AFz and an online reference at FT9. Electrodes were distributed around the scalp according to the International 10/20 system. Electrode impedance was adjusted to less than 15 kΩ before the first block and impedance levels were checked at the end of each block to ensure their optimal range. Continuous EEG signals were digitized with a Brain Vision actiCHamp plus amplifier at 500 Hz and recorded in Brain Vision Recorder. The EEG task was controlled using a custom-made Psychtoolbox-3 interface written in MATLAB (R2021b). The interface was used to deliver VIEWPixx TTL triggers to the EEG system to estimate stimulus onsets.

To minimize the presence of muscle artifacts, participants were seated in a comfortable chair with an adjustable headrest. They were instructed to refrain from moving as much as they could while the audio was being played and were offered small breaks between blocks to drink water and stretch their arms and legs. To minimize the presence of eye blink artifacts, participants were instructed to listen to the audios with their eyes closed and their performance was monitored with an infrared live feed camera. The duration of each block was approximately 15 min, resulting in an active task time of approximately 45 min. Total time per individual including setup, instructions, breaks, and the behavioral ratings task was under 2 h.

### EEG preprocessing

EEGs were preprocessed in MATLAB (R2021b) using a combination of custom-made and EEGLAB (Delorme and Makeig, 2004) functions. EEG data were offline re-referenced from the online reference to the averaged mastoids.

For neural tracking analyses, continuous EEGs were band-pass filtered between 1 and 8 Hz using a zero-phase 4th-order Butterworth. Eye movement and muscle artifacts were removed using ICA, implemented with the EEGLAB functions *pop_runica.m*, *pop_icalabel.m*, *pop_icflag.m* (artifact probability threshold = 0.9 for eye and muscle components), and *pop_subcomp.m*. The resulting EEGs were segmented into discrete epochs from stimulus onset to 500 ms post stimulus offset for neural tracking analyses. Neural tracking latencies were adjusted at the single-epoch level using a cross-correlation approach (e.g., see Zoefel and VanRullen, 2016) between the speech envelope and the EEG signal. For each epoch and each channel, we computed the (unnormalized) cross-correlation function:


$$R(\tau ,\text{channel})=\sum_{t}E(t)\;X(t+\tau )$$

where E(t) denotes the speech envelope, X(t) the EEG signal, and τ the time lag. Cross-correlations were computed using MATLAB’s *xcorr* function and evaluated for non-negative lags between 0 and 150 ms. The cross-correlation function R(τ,channel) quantifies the similarity between the envelope and the EEG signal as a function of channel and temporal lag. For each epoch and channel, latency was defined as the lag τ within the 0–150 ms window at which R(τ) reached its maximum value. This window was selected to prevent neural latencies preceding the stimulus onset or significantly exceeding the latency of the N1 component of the EEG. Because cross-correlation was used exclusively to estimate the lag corresponding to maximal similarity (i.e., the argmax over τ ) and not to interpret correlation magnitude, normalization was not required, as it would not affect the location of the peak lag. This alignment procedure accounts for trial-by-trial variability in neural response latency, resulting in more robust neural tracking estimates.

Epoch duration was then set to match the duration of the evoking stimulus, ensuring the same number of data points across neural and envelope oscillations. Epochs were then baseline-corrected using a 200 ms pre-stimulus interval and averaged for each unique combination of emotion (3 levels), sentence (10 levels), and channel (32 levels) within participants. Epochs with EEG amplitudes exceeding ±100 μV were excluded from the averaging.

For alpha power analyses, continuous EEGs were band-pass filtered between 0.1 and 30 Hz, cleaned using ICA, and segmented into epochs from stimulus onset to stimulus offset. Epochs were then baseline-corrected using a 200 ms pre-stimulus interval and averaged for each unique combination of emotion (3 levels), sentence (10 levels), and channel (32 levels) within participants. Epochs with EEG amplitudes exceeding ±100 μV were excluded from the average.

### Neural tracking analyses

**Figure 2:**
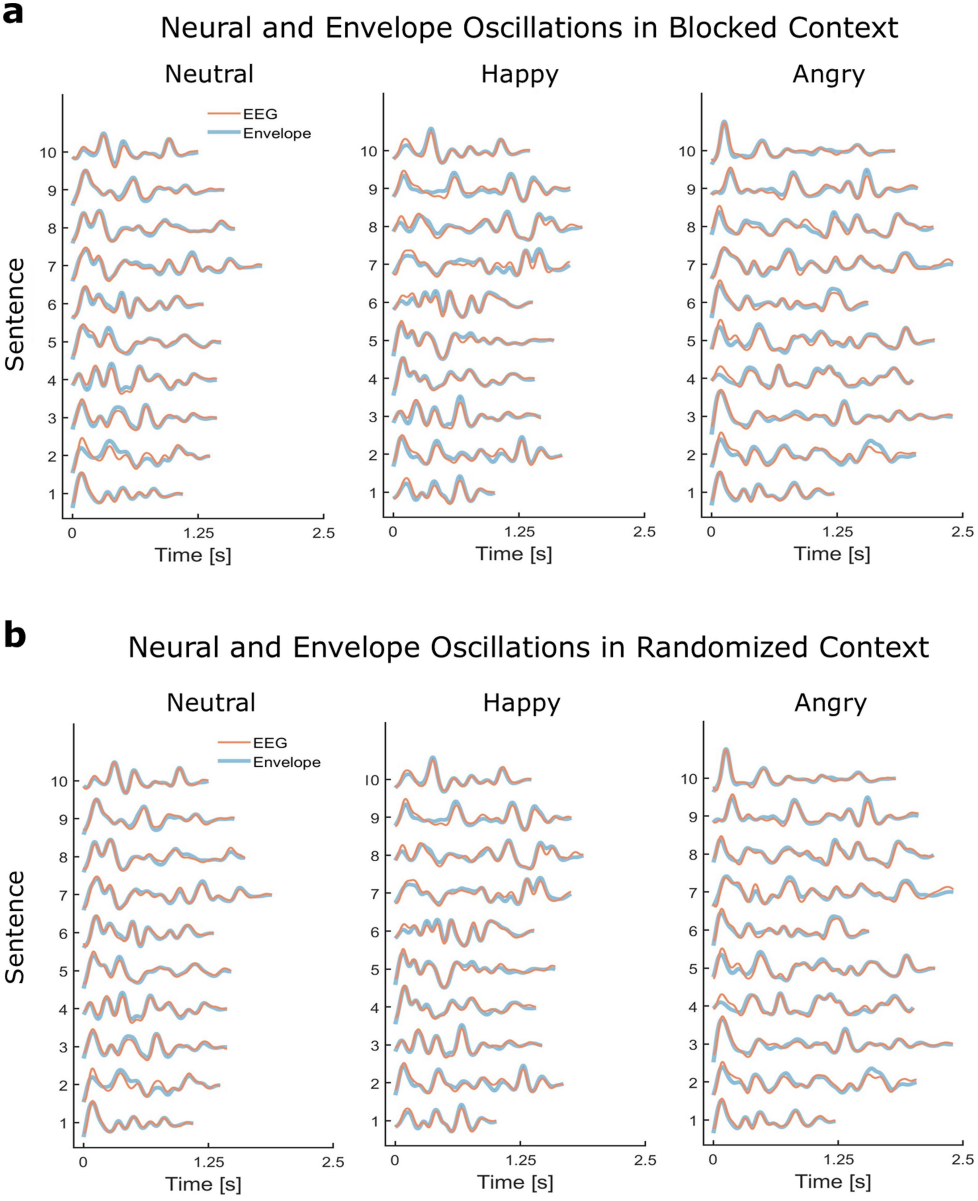
Synchronization between neural and envelope oscillations. Stimulus amplitude envelopes and grand-averaged evoked responses (*z*-scored units) when stimulus presentation was blocked by emotion **(a)** or randomized across emotions **(b)**.

Contemporary approaches like the Temporal Response Function (TRF) (Crosse et al., 2016; Lalor and Foxe, 2010) provide a powerful linear regression framework for modeling the relationship between continuous speech and the resulting neural responses over extended time scales, on the order of minutes. In the present study, we opted for a more direct sentence-level approach. Neural tracking was determined as the Pearson’s r correlation coefficient between the average EEG response and the amplitude envelope of the evoking stimulus. This approach resulted in quite robust mean neural tracking correlation coefficients (between 0.6 and 0.8; see the *Results* section and Figure 2) and was particularly well-suited for our goal of assessing the neural encoding of specific amplitude modulation patterns, as it captures the strength of the neural representation itself rather than the weights of a linear regression model.

Neural tracking coefficients were modeled using the following linear mixed-effects equation in R: tracking ~ emotion * context * channel + (emotion | participant) + (1 | sentence). The equation included: fixed effects by emotion (factor levels: angry, happy, neutral), stimulus presentation (factor levels: blocked, randomized), and channel (32 levels); by-participant random intercepts and random slopes for emotion; and by-sentence random intercepts. Models were fitted using the *lme4* package, and post-hoc pairwise comparisons were conducted using the *emmeans* package with Tukey adjustments, or FDR for channel-level analyses (32 levels).

To partialize the effect of envelope variability on the neural tracking of amplitude modulation, neural tracking coefficients were additionally modeled with the following linear mixed-effects equation in R: tracking ~ emotion * context * channel + envelope variability + (emotion | participant) + (1 | sentence). This model included envelope variability, defined as the standard deviation of the stimulus envelope, as a covariate to partialize the contribution of prosodic differences to neural tracking accuracy. Envelope variability was selected because it showed the strongest acoustic differentiation among emotional categories (see the *Results* section).

To investigate the effects of block ordering on neural tracking, neural tracking coefficients within the blocked stimulus presentation context were modeled with the following linear mixed-effects equation in R: tracking ~ emotion * block ordering + (emotion | participant) + (1 | sentence), where *emotion* denotes the emotion levels (angry and happy) that were counterbalanced across participants in the blocked context. The equation included: fixed effects by emotion (factor levels: angry, happy) and block ordering (factor levels: angry first, happy first); by-participant random intercepts and random slopes for emotion; and by-sentence random intercepts. Models were fitted using the *lme4* package, and post-hoc pairwise comparisons (block-level differences by emotion level) were conducted using the *emmeans* package with Tukey adjustments for multiple comparisons.

### Alpha power analyses

Alpha power was estimated using a wavelet convolution approach, with separate wavelets centered at each integer frequency within the alpha band (8–12 Hz) applied to each epoch. For each target frequency, EEG segments were convolved with a complex Morlet wavelet (6 cycles), and instantaneous power was obtained as the squared magnitude of the convolution result. Power values were then averaged across the entire segment to yield a single alpha power estimate per trial. Alpha power estimates were then averaged across epochs for each unique combination of emotion, sentence, and channel within participants.

Alpha-band activity was quantified during stimulus presentation and compared across emotions rather than being expressed relative to a pre-stimulus baseline (e.g., Hauswald et al., 2022). This choice was motivated by characteristics of the experimental design that may render the pre-stimulus interval an unreliable reference due to potential carryover effects from the preceding emotional context (Bradley et al., 1996; Brouwer et al., 2013; Verduyn et al., 2015). In addition, participants were instructed to close their eyes only during sentence presentation to minimize blink artifacts during neural tracking analyses, whereas eye state was not controlled during pre-stimulus intervals. Because eye closure robustly increases occipital alpha power, baseline-referenced suppression measures would be confounded by systematic differences in eye state. Accordingly, alpha power was analyzed during sentence listening and contrasted across emotional and presentation contexts, allowing condition-dependent differences in vigilance to be assessed under controlled stimulus and task conditions.

Mean alpha power values were modeled using the following linear mixed-effects equation in R: alpha power ~ emotion * context * channel + (1 | participant) + (1 | sentence). The equation included: fixed effects by emotion (factor levels: angry, happy, neutral), stimulus presentation (factor levels: blocked, randomized), and channel (32 levels); by-participant random intercepts and by-sentence random intercepts. Random slopes by participant for emotion were omitted because slope levels showed substantial collinearity (*r* = 0.72) and the model failed to converge. Models were fitted using the *lme4* package, and post-hoc pairwise comparisons were conducted using the *emmeans* package with Tukey adjustments for multiple comparisons.

## Results

### Acoustic differences between vocal emotions

Envelope features yielded clear acoustic differences among vocal emotions (see Figure 1c). Envelope mean was significantly higher (Kruskal–Wallis: *χ*^2^(2, 27) = 17.97, *p* < 0.001; see Table 1 for pairwise comparisons) for angry speech (*M* = 49 × 10^−4^ a.u., SEM = 0.8 × 10^−4^ a.u.) than for happy speech (*M* = 18 × 10^−4^ a.u., SEM = 0.2 × 10^−4^ a.u.) and neutral speech (*M* = 0.9 × 10^−4^ a.u., SEM = 0.1 × 10^−4^ a.u.). Envelope standard deviation was significantly higher (Kruskal–Wallis: *χ*^2^(2, 27) = 24.54, *p* < 0.001) for angry speech (*M* = 0.08 a.u., SEM = 0.004 a.u.) than for happy speech (*M* = 0.03 a.u., SEM = 0.006 a.u.), and for happy speech compared to neutral speech (*M* = 0.01 a.u., SEM = 0.001 a.u.). These statistical differences indicate that the three emotional speech categories exhibited important differences in their temporal structure, including differences in envelope height and envelope variability, with the latter providing a three-way distinction among vocal emotions.

**Table 1:** Acoustic differences between stimulus emotions (M = mean, SD = standard deviation).

Prosodic feature	Contrast	Statistic	Corrected *p*
Envelope M	Angry – happy	*z*(27) = 2.08	0.03
	Angry – neutral	*z*(27) = 3.52	<0.001
	Happy – neutral	*z*(27) = 1.44	0.19
Envelope SD	Angry – happy	*z*(27) = 1.97	0.04
	Angry – neutral	*z*(27) = 4.14	<0.001
	Happy – neutral	*z*(27) = 2.16	0.02
F0 M	Angry – happy	*z*(27) = 0.57	0.77
	Angry – neutral	*z*(27) = 3.44	<0.001
	Happy – neutral	*z*(27) = 2.86	<0.01
Syllable rate	Angry – happy	*t*(27) = −3.03	0.01
	Angry – neutral	*t*(27) = −4.08	<0.01
	Happy – neutral	*t*(27) = −1.05	0.55

Results for F0 were less consistent. F0 standard deviation did not significantly change across stimulus emotions (Kruskal–Wallis: *χ*^2^(2, 27) = 1.89, *p* = 0.38). In contrast, F0 mean was significantly higher (Kruskal–Wallis: *χ*^2^(2, 27) = 19.44, *p* < 0.001; see Table 1 for pairwise comparisons) for angry speech (*M* = 286.62 Hz, SEM = 8.19 Hz) and happy speech (*M* = 272.62 Hz, SEM = 8.19 Hz) compared to neutral speech (*M* = 207.77 Hz, SEM = 4.93 Hz). No significant difference in F0 mean was observed between angry and happy speech. These findings indicate that both angry and happy speech were produced with substantially higher F0 than neutral speech, while F0 variability did not differ much across emotions.

Finally, syllable rate (ANOVA: *F*(2, 27) = 8.98, *p* = 0.001; see Table 1) was higher for neutral speech (*M* = 5.51 Hz, SEM = 0.28 Hz) and happy speech (*M* = 5.13 Hz, SEM = 0.25 Hz) than for angry speech (*M* = 4.01 Hz, SEM = 0.23 Hz). However, no significant difference in syllable rate was observed between happy and neutral speech. Therefore, angry speech was produced at a slower speech rate than both neutral and happy speech.

### Perceptual ratings of stimulus valence and arousal

**Figure 3:**
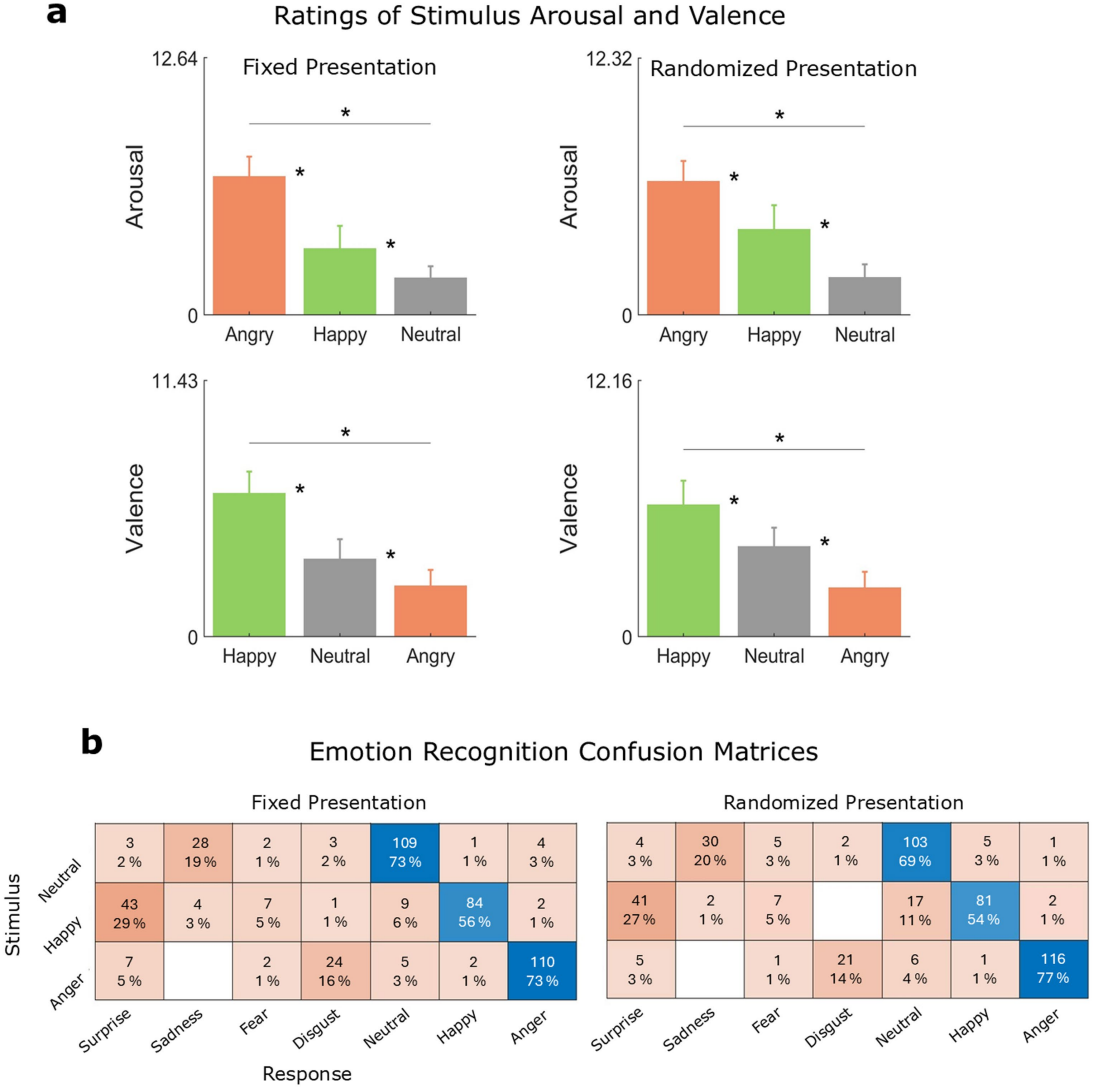
Behavior. **(a)** Perceived levels of stimulus arousal (top) and valence (bottom) by vocal emotion (M and SEM) and stimulus presentation order (fixed vs. randomized). Significant differences (*p* < 0.05) are indicated by a single asterisk (*). **(b)** Confusion patterns (counts and percentages rounded to the closest integer) between intended (speaker) and perceived (listener) emotions by stimulus presentation order.

In both stimulus presentation orders (fixed and randomized; see Figure 3a and Table 2), arousal ratings were higher for angry speech (fixed: *M* = 6.80, SEM = 0.13; randomized: *M* = 6.41, SEM = 0.13) than for happy speech (fixed: *M* = 3.26, SEM = 0.15; randomized: *M* = 4.12, SEM = 0.16), and higher for happy speech than for neutral speech (fixed: *M* = 1.82, SEM = 0.08; randomized: *M* = 1.80, SEM = 0.08).

**Table 2:** Emotion-level differences in arousal and valence ratings.

Feature	Contrast	Order	*t*	Corrected *p*
Arousal	Angry – happy	Fixed	11.47	<0.001
	Angry – neutral	Fixed	19.93	<0.001
	Happy – neutral	Fixed	4.29	<0.001
	Angry – happy	Randomized	7.43	<0.001
	Angry – neutral	Randomized	18.44	<0.001
	Happy – neutral	Randomized	6.89	<0.001
Valence	Angry – happy	Fixed	−14.68	<0.001
	Angry – neutral	Fixed	−4.64	<0.001
	Happy – neutral	Fixed	10.05	<0.001
	Angry – happy	Randomized	−13.99	<0.001
	Angry – neutral	Randomized	−7.64	<0.001
	Happy – neutral	Randomized	6.75	<0.001

Conversely, valence ratings were higher for happy speech (fixed: *M* = 6.39, SEM = 0.13; randomized: *M* = 6.25, SEM = 0.16) than for neutral speech (fixed: *M* = 3.46, SEM = 0.12; randomized: *M* = 4.28, SEM = 0.12), and higher for neutral speech than for angry speech (fixed: *M* = 2.28, SEM = 0.09; randomized: *M* = 2.33, SEM = 0.10). We observed no overall significant effect of stimulus presentation order in arousal ratings (|*t*| ≤ 1.18, *p* ≥ 0.10). However, when stimulus presentation order was randomized, neutral speech was rated with a marginally higher valence mean (*t* = 2.10, *p* = 0.044) compared to when stimulus presentation order was fixed.

### Recognition of vocal emotions

All three intended emotions were recognized well above the chance level (= 14.29% correct; see Figure 3b). In both stimulus presentation contexts (see Table 3), the percentage of correct categorization responses was significantly lower for happy speech (fixed: *M* = 56%, SEM = 4.07%; randomized: *M* = 54%, SEM = 4.08%) than for angry speech (fixed: *M* = 73.33%, SEM = 3.62%; randomized: *M* = 77.33%, SEM = 3.43%) and neutral speech (fixed: *M* = 72.67%, SEM = 3.65%; randomized: *M* = 68.67%, SEM = 3.80%). The comparatively lower recognition of happy speech reflects its perceptual overlap with surprise speech. Because “surprise” was available as a response option, some happy tokens were likely perceived as a pleasant or happy surprise, consistent with previous work (Cao et al., 2014; Lausen and Hammerschmidt, 2020; Pell et al., 2009).

**Table 3:** Emotion-level differences in recognition accuracy.

Contrast	Order	*z*	Corrected *p*
Angry – happy	Fixed	3.23	0.003
Angry – neutral	Fixed	0.13	0.99
Happy – neutral	Fixed	−3.10	0.005
Angry – happy	Randomized	4.33	<0.001
Angry – neutral	Randomized	1.74	0.18
Happy – neutral	Randomized	−2.69	0.019

No significant differences in correct emotion recognition were observed between angry and neutral speech in either stimulus presentation order. Additionally, we found no effect of stimulus presentation order on recognition accuracy when percentages of correct responses were collapsed across emotions (|*z*| ≤ 0.77, *p* ≥ 0.43).

We found similar confusion patterns across stimulus presentation orders. In both orders, neutral speech was most often confused with sadness, happy speech with surprise, and angry speech with disgust (see Figure 3b). The results of the chi-square test indicated that the distributions of categorization responses in the two stimulus presentation orders were not independent of each other (*χ*^2^(6) = 0.81, *p* = 0.99). This indicates that stimulus presentation did not significantly influence the perception of differences between emotions.

### Effects of vocal emotions on neural tracking

Amplitude modulation patterns were robustly tracked across emotional speech contexts (Figure 2), with a mean correlation coefficient of 0.72 (see Supplementary material for additional information on neural tracking mean and standard deviation by sentence, emotion, and stimulus presentation context).

**Figure 4:**
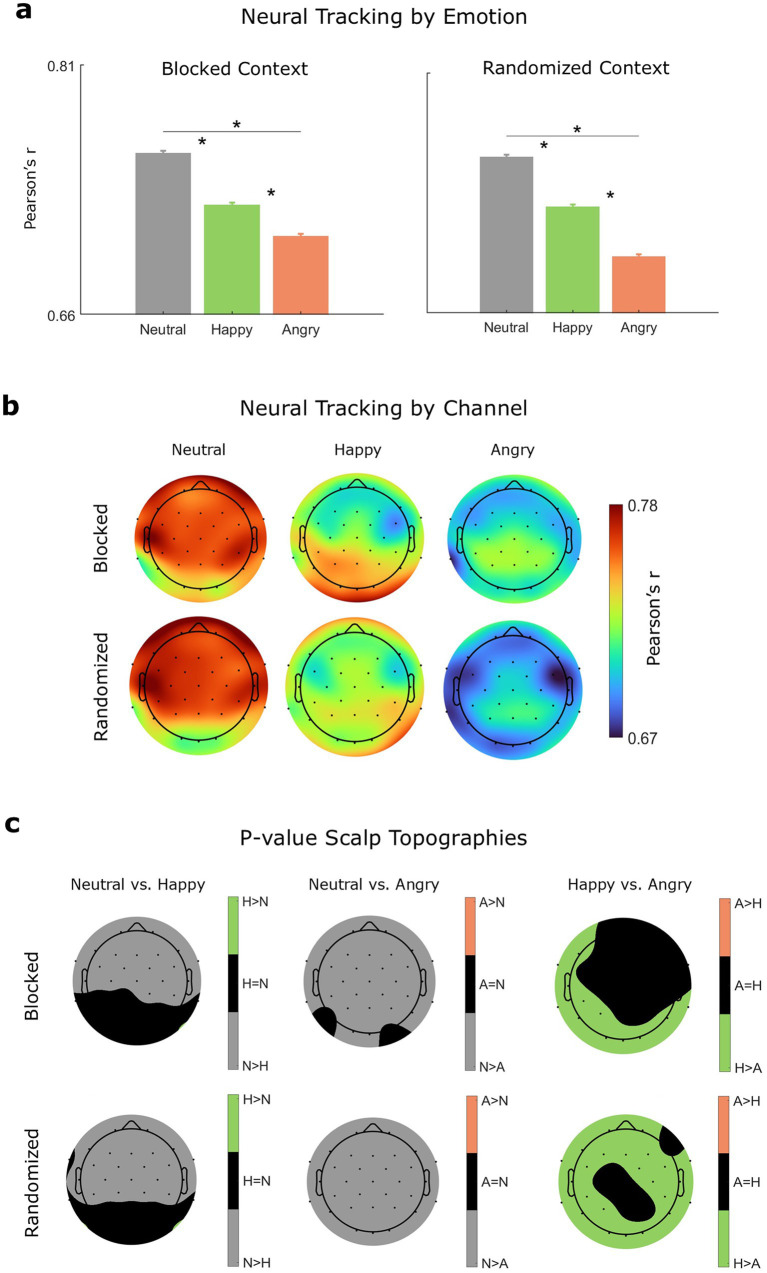
Neural tracking. **(a)** Neural tracking correlation coefficients by emotional speech context (M and SEM) for each stimulus presentation context. Significant differences (*p* < 0.05) are indicated by a single asterisk (*). **(b)** Mean neural tracking correlation coefficients by channel and stimulus emotion. **(c)** EEG channels conveying significant (*p* < 0.05) differences in neural tracking between stimulus emotions (A = angry, H = happy, N = neutral).

In both stimulus presentation contexts, neural tracking (see Table 4 and Figure 4a) was significantly more faithful for neutral speech (blocked: *M* = 0.7571, SEM = 0.0014; randomized: *M* = 0.7578, SEM = 0.0014) compared to happy speech (blocked: *M* = 0.7266, SEM = 0.0013; randomized: *M* = 0.7261, SEM = 0.0013) and angry speech (blocked: *M* = 0.7071, SEM = 0.0013; randomized: *M* = 0.6955, SEM = 0.0013), and for happy speech compared to angry speech.

**Table 4:** Emotion-level differences in neural tracking.

Contrast	Context	*z*	Corrected *p*
Angry – happy	Blocked	−2.74	<0.027
Angry – neutral	Blocked	−8.07	<0.001
Happy – neutral	Blocked	−4.44	<0.001
Angry – happy	Randomized	−4.50	<0.001
Angry – neutral	Randomized	−10.08	<0.001
Happy – neutral	Randomized	−4.44	<0.001

Emotion-level differences in neural tracking were also apparent at the level of individual channels (see Figures 4b,c), where neutral speech showed higher correlation coefficients across several central, fronto-central, and frontal channels, and happy speech conveyed higher correlation coefficients than angry speech across a fewer number of frontal, parietal, central or occipital channels. Notably, we did not observe major emotion-level differences in neural tracking driven by differences in stimulus presentation (across emotions: |*z*| ≤ 1.88, *p* ≥ 0.068), suggesting that the neural encoding of amplitude modulation was not strongly influenced by emotional context.

Notably, when envelope standard deviation was included as a covariate, neural tracking was no longer strongest for neutral speech. Instead, angry speech, which was the emotion conveying greater envelope variability, exhibited a more faithful tracking of amplitude modulation than both happy (across contexts: *t* ≥ 5.33, *p* < 0.001) and neutral speech (across contexts: *t* ≥ 5.59, *p* < 0.001). This suggests that the previously reported emotion-level differences were strongly driven by stimulus differences in amplitude variability and, to a lesser extent, by stimulus arousal.

Finally, we found no significant effect of block order on neural tracking (across emotions: |*t*| ≤ 0.67, *p* ≥ 0.51), indicating that whether the happy or angry block was presented first did not influence neural tracking.

### Effects of vocal emotions on alpha power

**Figure 5:**
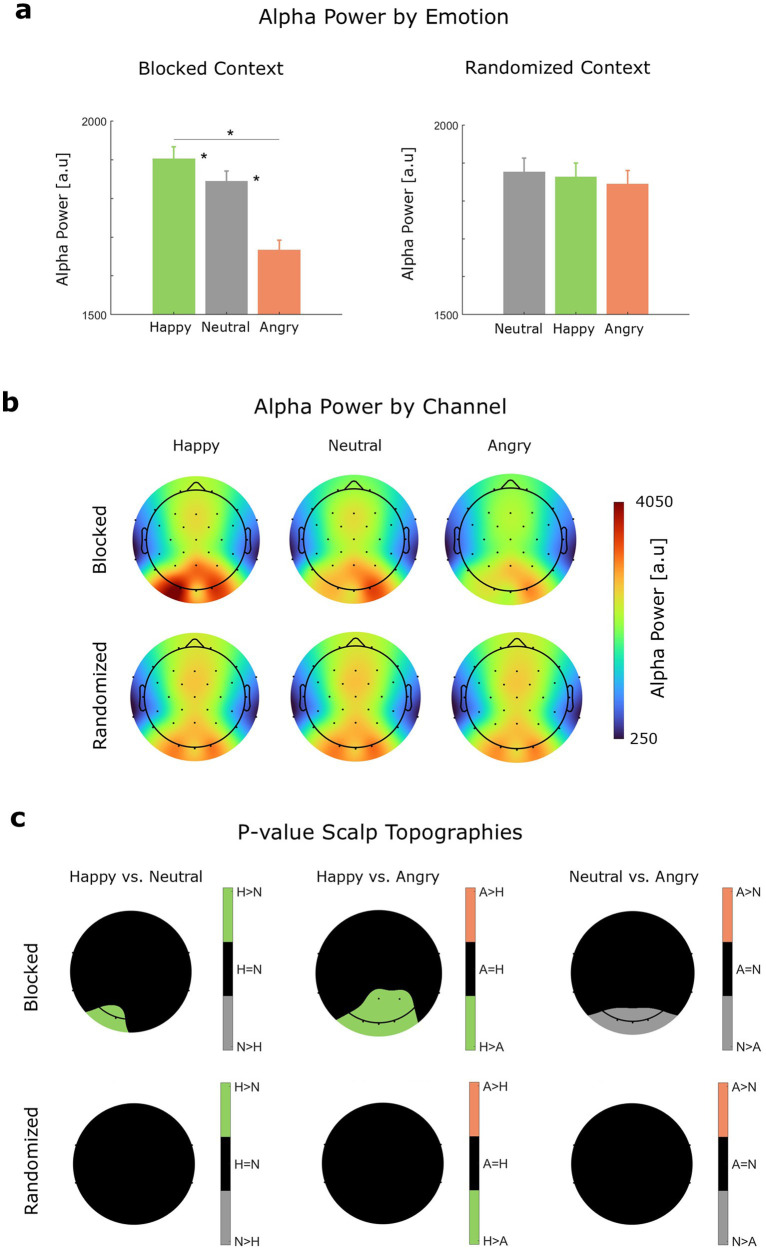
Alpha power. **(a)** Alpha power by stimulus emotion (M and SEM) for each stimulus presentation context. Significant differences (*p* < 0.05) are indicated by a single asterisk (*). **(b)** Alpha power by channel and stimulus emotion. **(c)** EEG channels conveying significant (*p* < 0.05) differences in alpha power between stimulus emotions (A = angry, H = happy, N = neutral).

As expected, alpha power was strongest over occipital channels (see Figure 5), consistent with prior work showing greater posterior alpha when participants close their eyes (Adrian and Matthews, 1934; Barry et al., 2007). Since participants were instructed to keep their eyes closed while listening to the audios, the observed occipital alpha may reflect inhibition of task-irrelevant visual processing.

In the blocked emotional context (see Table 5), alpha power was significantly lower (i.e., higher alpha desynchronization) for angry speech (*M* = 1666.36 a.u., SEM = 25.67 a.u.) compared to both happy speech (*M* = 1901.96 a.u., SEM = 31.17 a.u.) and neutral speech (*M* = 1844.22 a.u., SEM = 26.57 a.u.). Additionally, alpha power was significantly higher for happy speech than for neutral speech, although the effect was modest (*p* = 0.041). In the randomized context, no significant differences in alpha power were observed between angry (*M* = 1843.92 a.u., SEM = 36.24 a.u.), happy (*M* = 1863.03 a.u., SEM = 36.60 a.u.), and neutral speech (*M* = 1876.12 a.u., SEM = 36.66 a.u.). Thus, while there were no emotion-level differences in alpha desynchronization driven by differences in stimulus presentation (across emotions: |*z*| ≤ 0.45, *p* ≥ 0.65), stimulus presentation clearly affected the distribution of relative differences in alpha power within each context. Specifically, alpha power was associated with higher levels of vigilance for angry speech and lower levels of vigilance for happy speech, but only when stimulus presentation was blocked by emotion.

**Table 5:** Emotion-level differences in alpha power.

Contrast	Context	*z*	Corrected *p*
Angry – happy	Blocked	−9.84	<0.001
Angry – neutral	Blocked	−7.43	<0.001
Happy – neutral	Blocked	2.41	0.041
Angry – happy	Randomized	−0.79	0.70
Angry – neutral	Randomized	−1.34	0.36
Happy – neutral	Randomized	−0.54	0.84

## Discussion

We investigated the effects of vocal emotions on the neural processing of temporal speech patterns and internal processing states in listeners, comparing prolonged exposure to a single emotional tone with randomized exposure across emotions. Prior work has shown that vocal emotions can facilitate or disrupt processing depending on multiple factors. High-arousal stimuli, such as angry and happy speech (Russell, 1980; Verma and Tiwary, 2017) can increase attention and perceptual gain and therefore have the potential of enhancing the neural encoding of input properties (Aston-Jones and Cohen, 2005; Lakatos et al., 2008; Mather and Sutherland, 2011; Sara, 2009). However, angry and happy speech also exhibit greater amplitude variability (Laukka, 2005; Laukka et al., 2005; Lieberman and Michaels, 1962) that can potentially disrupt neural tracking by distorting the temporal structure of the speech signal. Whether neural tracking of emotional speech is enhanced or hindered, thus depends on the relative influence of these factors.

### Acoustic and behavioral findings

First, we identified the most significant prosodic differences between the three basic speaker’s emotions examined in the study (anger, happiness, and neutral emotion). Angry and happy speech showed greater envelope variability and higher F0 mean than neutral speech. These acoustic differences are consistent with prior work (Coutinho and Dibben, 2013; Laukka et al., 2005). Importantly, envelope variability showed a unique and clear three-way distinction between emotions, with angry speech showing the highest degree of envelope variability, followed by happy and then neutral speech (see envelope SD panel in Figure 1c).

Next, we investigated how our participants perceived the emotional speech stimuli. Each intended vocal emotion was associated with different perceived levels of stimulus arousal and valence. Here, we found a strong correspondence between envelope variability and arousal ratings, which covaried systematically across emotional categories. Angry speech was associated with greater amplitude variability and perceived arousal, whereas neutral speech was characterized by lower levels of both.

While intended emotions were recognized above chance, happiness was less accurately identified than angry or neutral speech and was frequently labeled “surprise.” This confusion pattern is consistent with the proximity of joy and surprise in the affective space when surprise is perceived as a pleasant surprise (Cao et al., 2014; Lausen and Hammerschmidt, 2020; Pell et al., 2009). Thus, the emotion recognition patterns documented in the present study are consistent with prior work.

Notably, neither emotion recognition nor arousal ratings were significantly influenced by emotional context. Arousal ratings were comparable across stimulus presentation contexts, and both recognition accuracy and confusion patterns remained highly consistent. Valence ratings showed only a marginal effect (*p* = 0.044), with angry speech receiving more positive ratings when stimulus presentation was randomized. This suggests that angry speech was perceived as slightly more negative when presented in the more predictable context. No additional context effects for valence ratings were observed for the other emotions. Overall, these findings indicate that stimulus presentation order exerted limited influence on how listeners perceived emotional speech features.

### Neural findings

Having established the distinctive acoustic and perceptual correlates of the emotional speech categories, we next examined how these categories influenced neural tracking and alpha power. We found a clear correspondence between emotion-level differences in envelope variability and neural tracking fidelity, with tracking being least faithful for angry speech, intermediate for happy speech, and most robust for neutral speech across stimulus presentation contexts. These findings suggest that acoustic features of speech stimuli, particularly envelope variability, may be more critical for neural tracking than the effect of speakers’ emotions on listeners’ processing states. If neural tracking were primarily driven by listeners’ vigilance states, one would expect a more systematic correspondence between alpha power and neural tracking across stimulus presentation contexts; but this was not the case. Furthermore, prior EEG evidence has shown that irregular prosodic patterns can disrupt the neural tracking of amplitude modulation in continuous speech (Falk et al., 2017; Kayser et al., 2015) in ways that are consistent with our findings, where greater changes in amplitude were more difficult to track.

Interestingly, when neural tracking was controlled for sentence-level differences in envelope variability, neural tracking was more robust for angry speech compared to happy and neutral speech. Given that angry speech displayed greater amplitude variability than happy and neutral speech, this result indicates that high-arousal stimuli may confer a modest yet quantifiable facilitatory effect, even in the presence of amplitude-driven processing challenges for neural tracking.

Whereas neural tracking was not substantially modulated by emotional context, alpha power was clearly sensitive to it. When stimuli were blocked by emotion, angry speech elicited greater alpha desynchronization (i.e., lower alpha power) than neutral or happy speech, particularly over occipital channels. In eyes-closed paradigms, occipital alpha suppression is commonly interpreted as an index of heightened vigilance or increased readiness (Barry et al., 2004, 2007; Wöstmann et al., 2020). This heightened vigilance may have contributed to the modest positive effects of high-arousal stimuli on neural tracking; however, these effects were negligible compared to the much stronger influence of envelope variability.

Taking an approach consistent with the Automatic Vigilance Hypothesis (Pratto and John, 1991), the selective alpha desynchronization observed for angry, but not happy, speech in the blocked condition is most naturally interpreted as reflecting enhanced vigilance toward negatively valenced, threat-related cues. Under this framework, attentional resources are preferentially allocated to aversive stimuli that signal potential harm, whereas positively valenced stimuli do not necessarily elicit the same automatic orienting response. Since both angry and happy speech are high in arousal, the comparatively weak alpha desynchronization for happy speech argues against a purely arousal-based mechanism and instead supports a vigilance-based interpretation. The lack of emotion-related alpha differences in the randomized condition further suggests that sustained exposure to a consistent affective context may be necessary for vigilance-related neural modulation to emerge, or that rapid alternation between emotional contexts may attenuate such effects.

The present findings suggest that alpha-band activity during emotional speech processing reflects differences in sustained vigilance states across emotional prosodies, rather than transient event-related suppression effects. In this study, alpha power was quantified during sentence listening and compared across intended emotions, capturing tonic modulations that persist throughout stimulus processing. This interpretation is consistent with frameworks distinguishing between phasic alpha dynamics, which reflect short-lived, event-locked changes relative to a reference baseline, and tonic alpha dynamics, which index broader state-related factors such as sustained attention, arousal, or cognitive control (Klimesch et al., 2007; Sadaghiani and Kleinschmidt, 2016). Within this state-based framework, lower alpha power during certain emotional speech contexts (e.g., angry relative to neutral or happy speech) is interpreted as reflecting higher sustained engagement or vigilance during listening, rather than a momentary release of inhibition time-locked to stimulus onset.

### Limitations and future work

Several limitations of the present study should be acknowledged. First, all stimuli were produced by a single speaker. This talker was selected because their portrayals of angry, happy, and neutral prosody were rated with high accuracy in the CREMA-D database, and our perceptual ratings corroborated their representativeness. Nevertheless, it remains uncertain whether the observed effects would generalize to speakers whose emotional expressions are less prototypical. Incorporating stimuli from a more diverse set of speakers in future work would allow for stronger conclusions about the generalizability of these findings.

A second limitation concerns our use of a between-subjects design to compare prolonged and randomized exposure to emotional speech. This approach was chosen to avoid potential confounds associated with mood induction, as sustained exposure to a single emotional tone can bias participants’ perceptual baselines. While this design reduced the risk of mood-induction carryover effects, it also limited sensitivity to subtle stimulus presentation differences compared to within-subjects paradigms.

A third limitation of the present approach is that it is specifically sensitive to tonic, state-related alpha dynamics during emotional speech processing and does not directly address phasic, event-related alpha suppression relative to a pre-stimulus baseline. As discussed above, task constraints, including emotional carryover effects and systematic differences in eye state between pre-stimulus and stimulus intervals, precluded the use of a stable baseline reference. Consequently, the present analyses cannot determine how emotional speech modulates transient, event-locked alpha responses at stimulus onset. Future studies employing designs with longer, emotion-neutral baselines and controlled eye-state conditions could dissociate phasic and tonic alpha dynamics more directly, allowing complementary insights into how emotional prosody shapes both immediate sensory responses and sustained attentional states during speech perception.

Finally, most of our participants identified as female, raising the possibility that the observed effects may reflect sex-specific patterns of emotional speech processing. Analyzing more balanced samples in future work will be critical for determining whether the present findings extend broadly across sexes and for assessing whether sex interacts with prosodic variability, perceptual judgments, or neural tracking in systematic ways.

## Data Availability

The datasets presented in this study can be found in online repositories. The names of the repository/repositories and accession number(s) can be found at: https://osf.io/j89bw/.
