# Supplementary material for: The effects of vocal emotions and emotional context on the neural tracking of speech envelopes and listeners’ vigilance states
Source: Front Hum Neurosci. 2026 May 8;20:1692628. doi: 10.3389/fnhum.2026.1692628 (PMC13194503; doi:10.3389/fnhum.2026.1692628)
Supplement: Supplementary file 1 [file Supplementary_File_1.docx]

**Supplementary Materials**

The supplementary figure below illustrates sentence-level neural tracking coefficients across emotions and conditions.


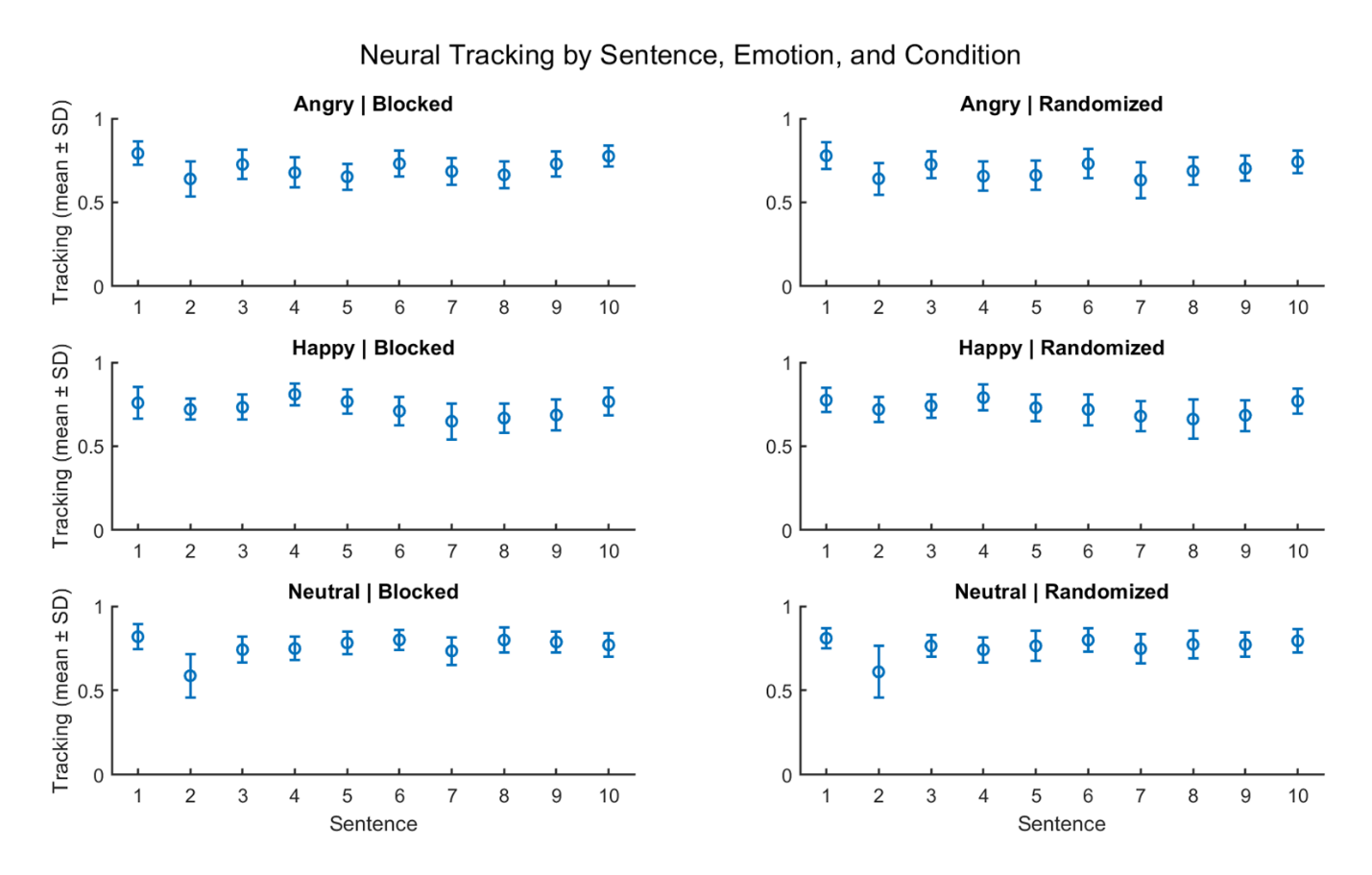


**Figure S1**. Mean neural tracking coefficients (± SD) for each sentence (1–10) are displayed separately for emotional category (Angry, Happy, Neutral) and presentation context (Blocked, Randomized). Tracking coefficients were computed after single-trial latency alignment. Consistent with the main analyses, tracking is generally stronger in the blocked condition and varies systematically across emotional categories. Error bars reflect variability across participants.
